# The Logic of Evolution: Review of the Logic of Chance by Eugene V. Koonin

**DOI:** 10.3389/fgene.2012.00135

**Published:** 2012-07-20

**Authors:** Richard D. Emes

**Affiliations:** ^1^School of Veterinary Medicine and Science, University of NottinghamNottingham, UK

What is the role of chance in evolution? How does this force influence the adaptionist view of evolutionary biology? Written by one of the leading researchers in the field of evolutionary and computational biology, Koonin (2011) provides both a personal narrative and informed perspective on the subject of biological evolution that is invigorating for the mind. This text provides both solid background information and synopsis of the current state-of-the-art within evolutionary theory but also thought provoking, novel ideas to challenge the experienced reader.

Although not for the more timid lay reader with text in places being very dense and acronym laden, summary boxes and synopses at the end of each chapter allow the reader to collect and consolidate essential ideas. Each chapter introduces novel ideas and although sometimes overly brief, the scientific research that has led to our current thinking. Whilst the chapters build from each other and would be best read in a single sitting (although prolonged as the book extends to over 500 pages including appendices on the “postmodern state of evolutionary biology” and “evolution of the cosmos and life”), the density of the text means that this is unlikely in all but the most dedicated of readers.

Early chapters of the text are aimed more at a general readership and provide detailed explanations and limitations of phylogenetic methods which are clear for those without prior understanding. These sections provide a comprehensive and stimulating review of the progress of evolutionary theory and experimentation. From Darwin and the central messages of the “origin of species” through an excellent introduction to the modern synthesis of the mid twentieth century to the “genomics revolution” of the latter twentieth and early twenty-first centuries.

By comparison to the world of physics, the methods and theory of comparative genomics and evolutionary biology are used to investigate the possibilities of universals in evolution. Such as, is the network topology of interacting genes scale free due to the duplication of genes interrupting interaction of ancestral genes? Later chapters return to the area for which Koonin is best known, the comparative genomics and evolution of prokaryotes. Here the personal narrative is clear again and the importance of considering the prokaryote and eukaryote worlds as separated by differences in evolutionary history is emphasized. The complexity of evolutionary behavior of prokaryote organisms is discussed at length. The role of population genetics and population structure is introduced and this theory is used to highlight that the relatively long term stability of eukaryote genes is not shared in the turbulent world of prokaryote evolution. This theme of the necessity for different thinking when considering eukaryotes, particularly the animal kingdom, and the prokaryotes is discussed at length in relation to phylogenomics and the tree of life. The limitations of the universal tree of life with bifurcating branches representing the clear relationships between species are also introduced. Whilst, the tree of life is a tempting entity that is often used as a shortcut when discussing the evolutionary relationship of ancient homologous genes, the more realistic forest of life with interconnecting web or net like structure which incorporates the near ubiquity of gene swapping or horizontal gene transfer between prokaryotes is introduced.

The theme of boiling down complex biological systems to physical universals is revisited when discussing the entropy or information content relative to genome size. The relative high entropy of “complex” eukaryote genomes which carry little information given their size is used as an argument against purely adaptionist thinking in the evolution of the genome. In contrast the non-adaptionist theory of genome evolution, reliant on the dominant effects of drift related to effective population size is used to highlight the prevalence of purifying selection in the prokaryotes. Here the author offers an excellent description and summary of the implications of work by Michael Lynch and John Conery on the non-adaptionist hypothesis of genome evolution. Eugene Koonin fluently introduces the theory of population genetics and its influence as a null hypothesis of genome evolution. Using the relatively simple explanations of Lynch and Conery, Koonin introduces the huge influence of drift on genome evolution. The author elegantly explains the process of how population dynamics influences genome size and complexity. Importantly the attractiveness and exceptions to these explanations are given using examples from comparative genomics. The book concludes with chapters on the hunt for and prediction of the form of the last universal common ancestor (LUCA), the origin of life and the problems with achieving understanding of this event. The author proposes that the term last universal common ancestral state (LUCAS) may be more accurate, and investigates the possibility of a non-cellular LUCAS.

With this text, Eugene Koonin does not shy away from discussion of difficult issues. It is clear Koonin has not set out to preach to the converted but rather has aimed to challenge commonly held thoughts. The introduction and review of Wrightian and Lamarkian evolution and the potentially contentious issue of “evolution of evolvability” for example is achieved by eloquent and persuasive examples. In conclusion the book is a demanding but rewarding read, bringing together accepted research themes and deliberately challenging areas. Whilst those familiar with evolutionary biology and the author's work will find it a fascinating read, it is also well worth the perseverance for non-specialists to appreciate the complexity and role of chance in evolution.
